# American Medical Association

**Published:** 1874-06

**Authors:** 


### Editorial.

### American Medical Association.

The twenty-fifth annual session of the American Medical Association was held in Detroit, commencing Tuesday, June 2d, 1874. The association assembled in Music Hall, and was called to order by the President, Dr. J. M Toner, of Washington, and opened by prayer by Rev. Bishop McCoskry, of Michigan.

The association was welcomed to Detroit by Dr. Wm. Brodie, Chairman of the Committee of Arrangements, who, in a short speech, offered the hospitality of the city to the members. The list of names as far as registered was then read, after which followed the annual address by the President.

Dr. Toner's address differed somewhat from the usual order pursued by his predecessors, and was listened to with marked attention. A large number of ladies and citizens were present at the opening session. The time and place of meeting of the several sections was then announced and the reports of special committees and several volunteer papers which were offered were referred to the appropriate sections.

In the afternoon the different sections were called to order by their presiding officers and at once proceeded to business.

In the section on Practical Medicine, Dr. N. S. Davis Chairman, Dr. L. D. Bulkley, of New York, read an interesting paper on the Nature and Treatment of Eczema, which called forth some remarks from several gentlemen present. Dr. P. J. Farnsworth, of Iowa, read a paper on the Therapeutic Uses of Ammonia, which was referred back to the author with permission to publish. The section on Obstetrics and Diseases of Woman, Dr. Parvin, Chairman, was occupied during its first session in inspecting some instruments presented by the Chairman, and in listening to a paper by Dr. Beck, of Fort Wayne, Ind., on a "New Theory of Generation." Dr. Beck's paper excited considerable discussion, which was participated in by Drs. White, Byford, Sims and Pallen. In the section on Surgery and Anatomy, of which Dr. S. D. Gross was Chairman, papers were read by Dr. Dunlap, of Springfield, Ohio, on Enchondrona over the Sternum, and by Dr. E. M. Moore, of Rochester, N. Y., on Epiphyseal Fracture of the Superior Extremity of the Humerus. Dr. Sayer presented a verbal abstract of a Report on Fracturs. Dr. Sayer's remarks excited as much discussion, probably, as any paper read during the meeting.

The section on Medical Jurisprudence and Chemistry met, and there being but a small attendance it adjourned without transacting any business.

The section on State Medicine and Public Hygiene, was presided over by Dr. A. N. Bell of Brooklyn. A paper by Dr. H. I. Bowditch, of Boston, on

State Boards of Health, was read by the Secretary. Drs. Kedzie, of Mich., Stewart, of Minnesota, and Bell, of Brooklyn; presented brief abstracts of papers on the same general topic Dr. Kedzie offered a resolution that the association petition Congress to form a National Sanitary Bureau, which was adopted after some discussion.

On the morning of the second day the association convened in the Opera House, the Music Hall having been found too small to accommodate all the members.

The usual preliminary business being transacted the report of the Judicial Council in relation to the revision of the Code of Ethics, was presented by Dr. N. S. Davis, Chairman of the Sub-Committee, to whom it was referred; the report, which we hope to present to our readers shortly, was unanimously adopted by the association. The amendments to the Constitution were next taken up and acted upon. The Constitution was so amended that in the future only State and Territorial Medical Societies and such District, County and City Medical Associations as are recognized by their State Societies; and the Medical Staffs of the Army and Navy are entitled to representation. Dr. J. M. Keller, of Louisville, presented a report on the Relative Rank of the Medical Staff of the U. S. Army, the report was received and the committee continued.

Dr. N. S. Davis then was introduced and delivered the annual address as Chairman of the Section on Practical Medicine, Materia Medica and Physiology. His address was well considered, and was listened to with marked attention. At its conclusion it was referred to the section above mentioned.

Dr. S. D. Gross, then delivered the annual address, as the Chairman of the Section Surgery and Anatomy. He took Syphilis as his subject and occupied full an hour and a half in the delivery of the address. It was listened to with fixed attention and received with applause. The address was referred to the section on Surgery, and the association adjourned.

In the afternoon the sections again assembled in their several rooms. In the section of Practical Medicine a paper on the Indigenous Medical Botany of West Virginia was presented from Dr. E. A. Hildreth, which was referred in the authors absence, to the President of the section with directions to report to the Committee on Publication. Dr. S. J. Deal's report on the cultivation of the cinchona tree was read and the committee continued.

Dr. W. M. Carpenter read a paper of considerable length on the Mechanism of the Encephalie Circulation by Dr. R. E. Vance, of N. Y., which was referred back to the author with permission to publish.

Dr. F. R Buckham, of Flint, Mich., read a paper on Uremia and its relation to Renal Diseases which was, after some discussion, referred to the Committee on Publication.

In the section on Obstetrics, Dr. Bontecue, of Troy, read a paper on Inverted Uterus, which was referred to the Publication Committee. Dr. Sims presented, on behalf of Dr. Scott of Canada, a new pessary which Dr. Scott explained to some extent; this excited a lively debate as to the value of pessaries, after which the section adjourned.

In the Section of Surgery the whole time was occupied by the discussion of Dr. Sayer's report on Fractures. Papers were read in the Section on Hygiene upon the influence of Drainage on the Public Health by Dr. Kedzie, Cabell and Bell, and on the Climatic Influences of Key West, by Dr. R. D. Murray of the U. S. M. H. Service, these papers were discussed and referred to the Commitee on Publication.

The session opened on the morning of Thursday, with a full attendance after the transaction of some routine bus ness and listening to the report of several Committees. Dr. Parvin, Chairman of the Section of Obstetrics, read his address on Uterine Hemorrhage and Transfusion, which was referred to his Section for consideration. Dr. A. N. Bell, Chairman of the Section on State Medicine and Public Hygiene, read his address on Waste of Life, which was received by a vote of thanks and referred to the Section on State Medicine for Consideration The reports from the Committees on Prize Essays and on Necrology and also of the Librarian were received and referred to the Publication Committee, after which the Association adjourned.

In the afternoon the Sections again assembled. Dr. Bulkley read a short paper in the Section on Practical Medicine, on a new Anti-Pruritic Remedy. The remedy which he presented consists of equal parts of Hydrate of Chloral and Gum Camphor rubbed up in Rose Ointment. He afterwards presented the remedy in a new form having rubbed the Chloral and Camphor in Glycerine. The address of the Chairman was then taken up, and after discussion was referred to the Publication Committee, and two Special Committees appointed to t ke into consideration its recommendations, and carry them into practical effect. Dr. Garish of N. Y., read a paper on Hydrophobia, which was referred back to the writer for publication in some medical periodical. Dr. E. W. Gray read a paper on the relation of Physiology to Practical Medicine, which was referred to the Publication Committee. Dr. E. Seguin of N. Y., gave a brief but interesting explanation of his method of Mathematical Thermometry, and a paper by Dr. J. J. Caldwell of Baltimore, on Electricity as a restorative agent in Narcosis and Asphyxia, was received and referred to a Special Committee after which the Section adjourned *sine die.*

In the Section on Obstetrics the Subjects of Ovariotomy and Transfusion were discussed, and the Chairman's address referred to the Publication Committee; the Section then adjourned.

The Section on Surgery spent some time in listening to an address by Dr. Geo. M. Beard on the "Uses of Electricity in Surgery," and in examining his instruments, after which it adjourned.

In the Section on Medical Jurisprudence and Chemistry, the Chairman, Dr.

E. Lloyd Howard read a paper on the "Legal Relatious of Emotional Insanity," and Dr. A. N. Talley one on the relations of "Psychology to Medicine," these were referred to the Publication Committee and the Section adjourned. The Closing Session of the Section on State Medicine was occupied in the Consideration of Miscellaneous matters, and the appointment of va ious Committees.

The last general session was held in the morning of Friday, June 5th. It was occupied by the consideration of the reports of the Nominating and Publication Committees, and of the Treasurer.

The Nominating Committee presented the following nominations, and their action was confirmed by the association:

*President*, Dr. W. K. Bowling, Nashville, Tenn.

*Vice-Presidents*, Drs. Wm. Brodie, Mich; J. J. Woodward, U. S. Army; H. W. Brown, Texas; and H. D. Didama of New York.

*Section on Practical Medicine, Materia Medica and Physiology*, Dr. Austin Flint of New York, Chairman; Dr. J. K. Bartlett of Wisconsin, Secretary.

*Section on Obstetrics and Diseases of Women*, Dr. W. H. Byford of Chicago, Chairman; Dr. S. C. Busey of Washington, Secretary.

*Section on Anatomy and Surgery*, Dr. E. M. Moore of Rochester, N. Y., Chairman; Dr. T. S. Latimer of Baltimore, Md., Secretary.

*Section on Medical Jurisprudence and Chemistry*, Dr. J. Cochrane of Mobile, Chairman; Dr. G. A. Moses of St. Louis, Secretary.

*Section on State Medicine and Public Hygiene*, Dr. H. I. Bowditch of Boston, Chairman; Dr. H. M. Bak r of Michigan, Secretary.

The place selected for the next annual meeting was Louisville, Ky.

The attendance at this meeting was very large, nearly five hundred members being present. Everything passed off smoothly and a large amount of work was transacted during the session. The citizens of Detroit seemed determined to make the members of the Association feel at ease and were profuse in their hospitalities. We do not think that a better meeting has been had for years.

